# The phyllochron of well-watered and water deficit mature peach trees varies with shoot type and vigour

**DOI:** 10.1093/aobpla/plx042

**Published:** 2017-09-01

**Authors:** Anna Davidson, David Da Silva, Theodore M DeJong

**Affiliations:** 1 Department of Plant Sciences, University of California Davis, One Shields Avenue, Davis, CA 95616, USA

**Keywords:** Epicormic, leaf appearance rate, phyllochron, plastochon, *Prunus*, *persica*, water deficit

## Abstract

The branch construction of trees is based on phytomers, repetitive subunits defined as node + leaf + axillary meristem + internode. The rate at which phytomers are added to a shoot is termed the phyllochron, which is determined by genetics, endogenous regulation and environmental conditions. The phyllochron is fundamental to understanding the growth of plants. Most phyllochron studies on woody species are of young plants under controlled conditions without consideration for different types of shoots that are present in mature trees. In this 2-year field study, we investigated seasonal patterns of phyllochron development on both proleptic and epicormic shoots of mature peach trees (*Prunus persica*) exposed to two irrigation treatments. One treatment was not irrigated until significant stress was detected via water potential by pressure bombing. In the second treatment trees were normally irrigated with ~5.84 cm of water each week to match evapotranspirational loss. Midday stem water potential readings were regularly collected to assess the level of water stress experienced by the trees. Measurements of individual leaves and their corresponding internodes were taken along tagged proleptic and epicormic shoots three times per week from the beginning to the end of two growing seasons. Leaf measurements were used to calculate the phyllochron. The phyllochron increased as the season progressed. This increase could not be explained by traditionally accepted effects of temperature or light. The more vigorous epicormic shoots added leaves significantly faster than proleptic shoots on trees in both water deficit and normally irrigated treatments. Additionally, epicormic shoots produced leaves with significantly greater leaf length and leaf area. Midday stem water potentials were more negative in trees in the water deficit treatments only after proleptic shoots stopped growing. The phyllochron did increase while leaf length and leaf area decreased on epicormic shoots of deficit irrigated trees in the 2010 growing season. The phyllochron of both shoot types gradually increased over the season, which was primarily due to an endogenous rank or shoot ageing effect. Differences between shoot types indicate that the phyllochron is variable among shoots on the same tree and is associated with shoot vigour. Water deficit increased the phyllochron and over all shoot growth rate.

## Teaser

The addition of new nodes to shoots is an essential mechanism for understanding and modelling canopy growth but surprisingly, there is very little research on the factors that control the phyllochron in tree species. It is often assumed that the phyllochron is regulated by temperature and is consistent among all shoot types. This research clearly indicates that this is not the case. This study shows differences in the phyllochron between different shoot types and water treatments that appear to be associated with rank effect rather than temperature.

## Introduction

Growth and development of plants involve interdependent processes resulting from the interaction of genotype and environment (Callaham 1962; Lambers *et al.* 2008). Development and growth are characterized by the repeated formation, expansion and subsequent senescence of basic units, called phytomers (Gray 1879). A phytomer comprises a node and the tissues and organs derived from it: a leaf, axillary bud(s) and internode. Also known as metamers (White 1979), phytomers are the building blocks used to construct plants and are the basic functional element of many functional–structural plant models (Allen *et al.* 2005). The time elapsed between the addition of new phytomers can be represented and easily measured by the appearance of leaves at new nodes (Pagès *et al.* 1996). The number of leaves that emerge per unit of time is termed leaf appearance rate (LAR). The inverse of LAR is termed the phyllochron, the time elapsed between the appearances of successive leaves on a stem (Wilhelm and McMaster 1995) and is most commonly measured in hours, growing degree days (GDD) or growing degree hours (GDH). Thus, shoot development can be conceived as the addition of successive phytomers, added to the shoots during each phyllochron.

In spite of considerable progress in understanding molecular processes that lead to the sequential initiation of new leaves (Reinhardt *et al.* 2003), the role of specific environmental parameters in the regulation of the phyllochron is still unclear. Temperature exposure is generally considered to be the main environmental factor influencing the phyllochron (Dennett *et al.* 1978; Rawson and Hindmarsh 1982) followed by photoperiod (Rawson and Hindmarsh 1982; Cousens *et al.* 1992; Rawson 1993; Kirby 1995), incident radiation (Bertero 2001) and water status (Silk 1980; Mathews *et al.* 1987). Nitrogen availability (Longnecker *et al.* 1994), salinity (Maas and Grieve 1990) and atmospheric carbon dioxide (Hofstra and Hesketh 1975; Rogers *et al.* 1984) have also been reported to affect the phyllochron, but to a lesser extent.

Most studies on environmental factors that influence the phyllochron have been conducted on annual plants under relatively controlled conditions and patterns of phyllochron behaviour derived from these experiments are used for subsequent modelling of plant or crop growth (Jones *et al.* 2003). The current study was directed towards understanding the factors that influence the phyllochron of shoots of peach trees in order to develop more accurate models of the growth of canopies of mature field-grown trees.

Like most fruit trees, peach trees (*Prunus persica*) are composed of several shoot types that are botanically distinct from one another and display notably different behaviours within the canopy. The two main shoots in peach are termed proleptic and epicormic. Proleptic shoots grow from buds that have overwintered (Wilson 2000; Costes *et al.* 2006) and exhibit ‘preformed growth’, meaning shoot organs form within the dormant vegetative bud that will later give rise to a proleptic shoot (Gordon et al. 2006). At the time of spring bud break there are ~10 leaf primordia per vegetative bud (Gordon *et al.* 2006). Half of these are formed during the dormant period, the number being influenced by temperature during dormancy, with cold temperatures favouring the production of leaf primordia (Loiseau *et al*. 2001). Nodes formed subsequent to bud break of a proleptic shoot are ‘neoformed’, meaning the formation of additional leaves is dependent upon current season’s conditions.

In contrast, epicormic shoots grow vigorously from preventitious buds consisting of latent meristematic tissue located under the bark (Fink 1983; Wilson and Kelty 1994). Epicormic shoots are entirely neoformed; all of their nodes are dependent upon current season conditions. Epicormic shoots often grow in response to severe pruning or limb damage. These shoots are less productive in terms of fruiting and often shade out the important fruit-bearing proleptic shoots.

It is generally understood that epicormic shoots are more vigorous and grow faster than proleptic shoots. However, it is not known whether these two shoot types have different phyllochron behaviour. In addition, the preformed vs. neoformed growth of proleptic shoots adds an additional layer of complexity as well as the potential for different phyllochron behaviours.

The few existing phyllochron studies on perennial fruit species have included kiwifruit (Cieslak *et al.* 2011), grapevine (Schultz 1992), coconut (Mialet-Serra *et al.* 2008), oil palm (Legros *et al.* 2009) and peach (Kervella *et al.* 1995; Pagès *et al.* 1996; Davidson *et al.* 2015). Pagès et al. (1996) found that metamer emergence rate in peach decreased according to branching order and by the location of insertion along the axis of young potted trees. However, it was not shown if the phyllochron differs between different shoot types in mature peach trees. Thus, one objective of this research was to determine if the phyllochron differs between proleptic and epicormic shoots of mature field-grown peach trees.

It is well established that water availability is an important factor for controlling vegetative growth (Berman and DeJong 1996; Behboudian and Mills 1997; Solari *et al.* 2006) and fruit growth (Berman and DeJong 1996; Génard and Huguet 1996). Seasonal effects of temperature and water relations on vegetative growth are the integrated results of many daily growth events (Berman and DeJong 1996; Basile and DeJong 2003; Solari *et al.* 2006). These short-term interactions, when scaled over weeks to months, are important determinants of seasonal carbon partitioning trends. Solari *et al.* (2006) investigated daily responses of vegetative growth to manipulation of water status of peach trees grown on different rootstocks in the field and found that relative shoot extension growth rate was linearly correlated with midday stem water potential. While it is clear that even mild plant water stress can limit expansive shoot and fruit growth in peach, less is known about its effects on the phyllochron.

Although the phyllochron is fundamental to understanding vegetative growth, there have been few field-based studies in woody perennials. For modelling purposes, we wished to know how the phyllochron in peach trees varies over the course of the season and how it differs between shoot types that constitute the canopy. Because water has been previously shown to affect shoot growth, our second objective was to observe the effects of water deficit on the addition of phytomers. In this 2-year field study, we tracked the phyllochron of selected proleptic and epicormic shoots on trees grown in well-watered and water deficit irrigated treatments during two growing seasons.

## Methods

This research was conducted during the 2010 and 2011 growing seasons at the UC Davis Wolfskill Experimental Orchards in Winters, California. Four-year-old and subsequently 5-year-old trees of *P. persica* ‘Lorrie May’ (unreleased) grafted on *P. persica* ‘Controller 9’ rootstock growing in a sandy clay loam soil. Trees were spaced 1.83 m apart in the row with 5.18 m between rows and trained to the Kearney Agricultural Center perpendicular-V system (KACV) (DeJong *et al.* 1994).

### Experimental design

Two north-south oriented rows of trees (in the middle of a total of 11 rows) were organized into a randomized complete block design with three blocks, two treatments per block; well-watered and water deficit, and three replications (trees) per block per treatment for a total of nine trees in each irrigation treatment, or 18 trees total. Two proleptic and two epicormic shoots located at breast height were randomly selected from both the west- and the east-facing sides of the trees. Therefore, there were a total of 18 tagged epicormic shoots followed in the well-watered treatment and 18 tagged shoots in the water deficit treatment. Likewise, there were a total of 18 tagged well-watered proleptic shoots, and 18 water deficit proleptic shoots, for a total of 72 tagged shoots. If the shoots became damaged or ended growth uncharacteristically early, they were replaced by nearby shoots of the same type so that a consistent number of shoots were being monitored at all times. Shortly after initial fruit set, all of the fruits were removed from the trees in order to assess optimal vegetative growth unimpaired by crop load. Nitrogen was applied twice per year, 112 kg ha^−1^ in February and 56 kg ha^−1^ in September.

### Irrigation regime

Following the depletion of winter rains, normally irrigated trees were watered an average of 5.84 cm approximately every week using micro sprinklers. Irrigation was scheduled by employing the soil water balance method where the inputs were residual soil moisture and precipitation and the output was estimated evapotranspiration (ETc). Run-off and deep percolation were assumed to be negligible. Crop evapotranspiration was determined by multiplying reference ET (obtained by the on-site CIMIS weather station (California Irrigation Management Information System, http://www.cimis.water.ca.gov/)) by a crop coefficient given in FAO 56 (Allen *et al.* 1998). Water deficit was achieved by entirely plugging the micro sprinklers of selected blocks of trees. Plant water status was regularly monitored from 8 June 2010 until 16 September 2010 and from 4 May until 18 August 2011 by taking midday stem water potential (Ψ_ST_) measurements on each tree using a pressure chamber as described by McCutchan and Shackel (1992).

### LAR measurements

To assess leaf growth rates and the phyllochron, incremental measurements of every leaf growing on selected shoots were made using a metric ruler. These repeated measurements were made three times per week from 5 April to 29 September 2010 and from 13 April to 17 August in 2011. Hourly temperatures were recorded by two HOBO data loggers (Onset Computer Corporation, Bourne, MA, USA) located in the orchard and confirmed by the local CIMIS weather station located on-site.

### Data analysis

Leaf lengths recorded in the field were imported into a database, post-processed and analysed using Python 2.7 (http://www.python.org/) and matplotlib library (http://matplotlib.org/). When taking field measurements of very small leaves, it was impossible to capture the exact initiation point of the leaf without imposing damage. Additionally, the appearance of leaves sometimes occurred between days of data collection. Therefore, leaf appearance was normalized to the time when a new leaf was 2.0 cm long. This was achieved by first plotting the incremental measurements of each leaf to create a leaf growth rate curve by fitting the individual leaf length data to a classical growth curve using the Gompertz model (Gompertz 1825) as previously described by Davidson *et al.*, 2015. With the normalized leaf appearance times, the time interval between two successive leaves in thermal time was estimated using a modified GDH scale (Davidson *et al.* 2015) that was based on Richardson *et al*. (1975). The GDH model was linear on both sides of a plateau-shaped optimum. The base temperature (*T*_B_) was 4 °C, the critical or maximum temperature (*T*_C_) was 40 °C. The heat accumulation between the optimal temperatures of 18 °C and 32 °C (*T*_O1_ and *T*_O2_) was flat. Therefore, when the current temperature (*T*_H_) was below *T*_B_ or above *T*_C_, nothing was added to the GDH accumulation. When *T*_B_ < *T*_H_ < *T*_O1_, *T*_H_ − *T*_B_ was added to GDH accumulation. When *T*_O2_ < *T*_H_ < *T*_C_, (*T*_C_ − *T*_H_) * (*T*_O1_ − *T*_B_)/(*T*_C_ − *T*_O2_) was added to the GDH accumulation. When *T*_O1_ ≤ *T*_H_ ≤ *T*_O2_, *T*_O1_ − *T*_B_ was added to GDH.

A grand mean phyllochron for each treatment was calculated by taking the mean phyllochron for each tree (two shoots per tree) and calculating the mean of all the trees within a treatment (nine trees per treatment) in a given 10-day time interval. To identify shoot type–water treatment–time interactions, an analysis of variance with 1 SE from the mean was calculated using JMP version 10 (SAS Institute Inc., Cary, NC, USA, 1989–2010).

## Results

The phyllochron for epicormic shoots was always significantly less than the phyllochron of proleptic shoots for a comparable time period in both the 2010 and 2011 seasons (Table 1). In other words, leaves were produced along a shoot on average 23 % (2010) and 36 % (2011) faster on epicormic compared to proleptic shoots. Additionally, leaf length and leaf area were both greater for epicormic leaves compared to proleptic leaves in both seasons. Mean leaf areas were 20 % and 30 % greater on epicormic compared to proleptic shoots in 2010 and 2011, respectively. Generally, in 2011, there were greater differences in the phyllochron, leaf length and leaf area between shoot types than in 2010.

**Table 1.** Mean phyllochron, mean leaf length and mean leaf area of proleptic and epicormic shoots grown in 2010 and 2011 in both well-watered and water deficit treatments (mean + SE).

	2010			2011		
	Proleptic	Epicormic	*P*-value	Proleptic	Epicormic	*P*-value
Phyllochron (GDH)	857.55 ± 32.918	663.08 ± 29.142	<0.0001*	988.01 ± 30.18	633.41 ± 29.363	<0.0001*
Leaf length (cm)	16.73 ± 0.182	18.82 ± 0.168	<0.0001*	15.66 ± 0.320	19.08 ± 0.318	<0.0001*
Leaf area (cm^2^)	58.29 ± 1.288	72.88 ± 1.193	<0.0001*	52.68 ± 2.039	75.76 ± 2.027	<0.0001*

*Represents significant differences.

**Figure 1.**
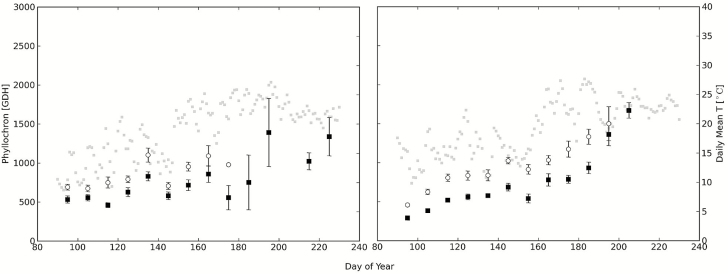
Phyllochron for all epicormic (black squares) and proleptic shoots (white circles) bundled for all treatments over the course of the field season in 2010 (left) and 2011 season (right) plotted in GDH. Error bars represent the standard errors of the mean. Mean daily temperature is plotted as grey squares in the background.

The phyllochron of both shoot types fluctuated during the season, more so in 2010 than in 2011 (Fig. 1). Generally, there was an upward trend or slowing of the phyllochron as the season progressed even though the mean daily temperatures increased over the same period. The trend of an increasing phyllochron over the season was more pronounced during 2011 than in 2010. In 2010, the proleptic shoots stopped growing at day 180, which is typical for these types of shoot, which have determinate growth (DeJong *et al.* 2012).

**Figure 2.**
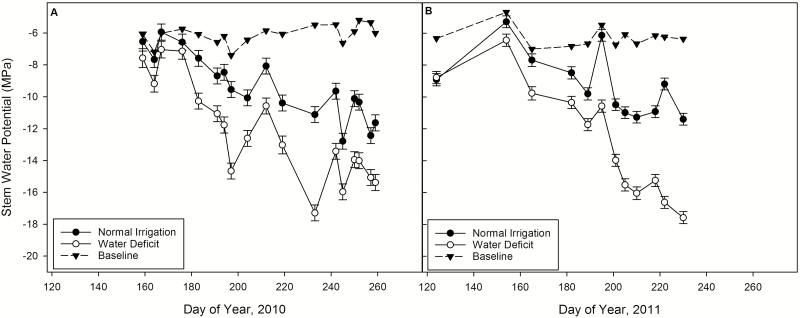
Midday stem water potential readings for the 2010 (left) and 2011 (right) season in normal and water deficit irrigation treatments compared with estimated baseline values that would represent no water stress. Error bars represent the standard errors of the mean.

The 2009/10 and 2010/11 seasons were exceptionally wet for California. Therefore, it was difficult to achieve a substantial early season water deficit in deficit-irrigated trees. From September 2009 until May 2010 total precipitation was 59.1 cm. From September 2010 until May 2011 total precipitation was 61.2 cm. Differences in midday stem water potential between treatments were not observed until June in both years (Fig. 2). While tree midday stem water potentials were always lower in the water deficit treatment, these differences were apparently not great enough to affect the phyllochron until August, long after proleptic shoots had stopped growing.

**Figure 3.**
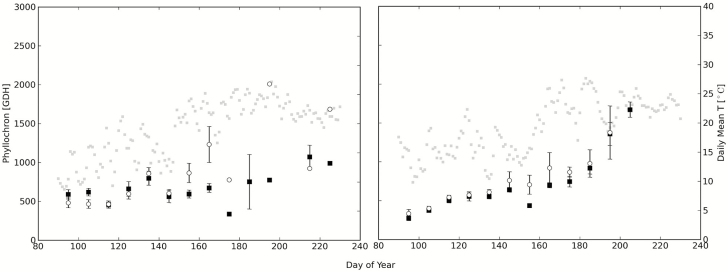
Estimated mean phyllochron plotted in 10-day periods for epicormic shoots over the course of the 2010 (left) and 2011 (right) field season in normal (black squares) and water deficit treatments (white circles). Error bars represent the standard errors of the mean. Mean daily temperature is plotted as grey squares in the background.

The significant effect of water deficit on the phyllochron was observed for epicormic shoots growing in 2010 (Table 2; Fig. 3). Mean leaf length and mean leaf area were also significantly less in water deficit treatments. The phyllochron along proleptic shoots with related mean leaf length and mean leaf area was generally unaffected by water deficit in 2010. The mean phyllochron of epicormic shoots grown in water deficit (925.9 GDH) was longer than the phyllochron of proleptic shoots grown in either irrigation treatment (832.8 GDH in well-watered and 877.2 GDH in water deficit) (Table 2).

**Table 2.** Mean phyllochron, mean leaf length and mean leaf area for proleptic and epicormic shoots grown in well-watered and water deficit treatments in 2010 (mean + SE).

2010	Epicormic			Proleptic		
	Well watered	Water deficit	*P*-value	Well watered	Water deficit	*P*-value
Phyllochron (GDH)	710.54 ± 59.258	925.91 ± 59.257	0.0143*	832.81 ± 48.055	874.20 ± 48.055	0.553
Leaf length (cm)	19.37 ± 0.241	17.22 ± 0.256	<0.0001*	16.57 ± 0.443	16.427 ± 0.469	0.828
Leaf area (cm^2^)	76.85 ± 1.822	61.729 ± 1.933	<0.0001*	57.484 ± 2.842	56.157 ± 3.009	0.755

*Represents significant differences.

During the 2011 season, only minor water deficit effects were observed on the phyllochron of epicormic shoots around days 150–170 but these effects were not large enough to lead to differences in the overall mean phyllochrons of either shoot type (Table 3). However, mean leaf length and mean leaf area of epicormic shoots were both significantly greater in the well-irrigated treatments.

**Table 3.** Mean (+ SE) phyllochron, mean leaf length and mean leaf area for proleptic and epicormic shoots grown in well-watered and water deficit treatments in 2011.

2011	Epicormic			Proleptic		
	Well watered	Water deficit	*P*-value	Well watered	Water deficit	*P*-value
Phyllochron (GDH)	712.05 ± 47.193	742.75 ± 63.726	0.1873	992.75 ± 41.31	980.44 ± 38.289	0.833
Leaf length (cm)	19.25 ± 0.367	17.25 ± 0.394	0.0085*	14.961 ± 0.716	16.262 ± 0.705	0.2263
Leaf area (cm^2^)	77.18 ± 2.530	63.50 ± 2.702	0.0080*	48.35 ± 4.336	56.351 ± 4.28	0.2202

*Represents significant differences.

## Discussion

Epicormic shoots are botanically distinct from proleptic shoots and display notably different behaviour within the canopy. Correspondingly, the phyllochron of epicormic shoots was significantly less than for proleptic shoots. This was complemented by longer mean leaf lengths and greater mean individual leaf areas. The epicormic shoots in our study also grew later into the season and stopped elongating between August and September (which may account for an increase in SD during that time) while proleptic shoots typically stopped growth by mid- to late June. With the additional growing time and their vigorous nature, epicormic shoots typically put on about twice as many phytomers as proleptic shoots. It is still unclear why proleptic shoots cease growth so much earlier than epicormic shoots but it has been suggested that proleptic shoots of peach trees are determinate (DeJong *et al.* 2012), whereas epicormics shoots appear to be indeterminate.

Most studies find that the LAR or the phyllochron are constant under constant environmental conditions such as in growth chambers as observed in potato (Cao and Tibbitts 1995) and sunflower (Villalobos and Ritchie 1992) and peach (Davidson *et al.* 2015). This field study, however, showed a fluctuating phyllochron and a general upward trend or increase of the phyllochron over the season no matter what the shoot type, treatment or year (Figs 1 and 3). The point of employing a thermal time (GDH) model to measure the phyllochron is to account for the effects of temperature. The phyllochron is generally thought to decrease with increasing temperatures. Thus, the seasonal upward trend in the phyllochron was not likely due to seasonal temperature increases. Even if temperature were partially effective, it could be expected that increasing temperatures early in the season when average temperatures are relatively cool would decrease the phyllochron rather than increase it. Additionally, there were no clear relationships between phyllochron and seasonal changes in daily irradiance. Daily irradiance generally increased as the days lengthened until day of year 180 and then subsequently decreased as the days shortened. Despite this pattern of early increase and later increase in total irradiance the phyllochron generally increased over the entire season. Therefore, we suspect that gradual increase of the phyllochron over the season is likely due to an endogenous rank effect, or to shoot ageing (Davidson *et al.* 2015). Kervella *et al.* (1995) and Pagès *et al.* (1996) also reported a slowing of the phyllochron and metamer emergence rate, respectively, over the course of the season in young potted peach trees grown outdoors. A slowing of the phyllochron over the season was also observed in grapevine (Schultz 1992).

We wished to detect a change in the phyllochron between the preformed and neoformed growth periods of proleptic shoots. However, since the preformed growth may only involved the first 10–11 nodes (Gordon *et al.* 2006) and there was a gradual increase in the phyllochron as the season progressed, it was not possible detect differences in the rate of leaf appearance separately for the preformed and neoformed parts of proleptic shoots.

Due to heavy rains during the previous winters, the efficient water holding capacity of the soil at the experimental site, and their shorter growing season of just 3 months, proleptic shoots finished growing before growth could be affected by water deficit. Differences in stem water potential did not occur until mid-June to late July (Fig. 2). Therefore, our experiments did not determine the effects of water deficit on the phyllochron, leaf length or leaf area of proleptic shoots (Table 2).

In 2010, water deficits were sufficient to slow the phyllochron of the epicormic shoots by an average of 23 % (Table 2; Fig. 3). These results compliment the previous study by Berman and DeJong (1996) who found that under reduced irrigation, the length of the stem elongation zone and total daily stem growth were significantly decreased and highly correlated with midday stem water potential. The epicormic shoots in both years of our study also had significantly greater leaf length and leaf area (Table 2). Silk (1980) also reported greater leaf size under well-irrigated conditions for cantaloupe. It is interesting to note that mean leaf size on epicormic shoots was significantly reduced by water deficit treatments in both years but the same differences were not registered with differences in the phyllochron. Perhaps leaf growth is more sensitive to water deficits than is the phyllochron, since the differences in stem water potential between the two irrigation treatments were significant in both years but less in 2011 than 2010.

The differences in leaf size on proleptic shoots compared to epicormic shoots were similar to the differences in the mean phyllochrons of the corresponding shoot types. This along with the water deficit responses indicates that the phyllochron of peach shoots may be related to the vigour of the shoot. It is generally known that the growth rate of the primary axes of growing shoots decreases as the season progresses (DeJong *et al*. 1987) and the pattern of increasing phyllochrons over the season appears to follow a similar pattern. Thus, unlike the notion of modelling the phyllochron based on temperature, photoperiod or irradiance that is often used for estimating the phyllochron of annual plant growth (Bertero 2001; Jones *et al.* 2003), regulation of the phyllochron in trees like peach is much more complex and may involve many of the previously known factors that have been reported to influence the phyllochron, as well as factors related to shoot vigour such as shoot type and node rank or seasonal timing.

## Conclusions

The phyllochron of epicormic shoots was less than for proleptic shoots and this was associated with greater mean individual leaf area and leaf length for epicormics shoots. Epicormic growth extended late into the season, long after proleptic shoots stopped their growth. The phyllochron for both proleptic and epicormic shoots fluctuated over the season but generally increased as the season progressed. This gradual increase appeared to be primarily due to an endogenous rank or shoot ageing effect. While there were differences in midday stem water potential, minimal differences were observed for the phyllochron between irrigation treatments except for epicormic shoots late in the 2010 season. The imposed water deficit also decreased individual leaf length and leaf area on epicormic shoots in 2010. Differences in the phyllochron for epicormic vs. proleptic shoots and the increasing phyllochron over the season suggest that the phyllochron is associated with shoot vigour. Importantly, it is clear that models of tree growth cannot rely on simple thermal time models of phyllochron behaviour for realistic simulation of canopy growth but must incorporate complex models to account for the behaviour of different shoot types and the fact that the phyllochron increases as the season progresses in a manner that does not appear to be directly related to temperature.

## Sources of Funding

None declared.

## Conflicts of Interest

None declared.
